# A complex case of acute abdomen

**DOI:** 10.1186/s12245-025-00954-9

**Published:** 2025-08-11

**Authors:** Mohammed Khalid Alageel

**Affiliations:** 1 Department of Emergency Medicine and Critical care, College of Medicine, King Saud University, Riyadh, Saudi Arabia; 2 Department of Emergency Medicine, University of British Columbia, Vancouver, Canada

**Keywords:** Appendectomy, Intestinal malrotation, Left-sided appendicitis, Pregnancy, Inflammatory bowel diseases, Ustekinumab, Emergency medicine

## Abstract

**Introduction:**

Acute abdomen during early pregnancy poses a diagnostic challenge, especially in patients with underlying chronic gastrointestinal diseases.

**Case report:**

A 36-year-old woman with Crohn’s disease and a confirmed 6-week intrauterine pregnancy presented with diffuse abdominal pain. Initial investigations, including MRI and ultrasound, were inconclusive. Diagnostic laparoscopy revealed malrotation of the large bowel and a perforated appendix, which was successfully managed with appendectomy and lavage.

**Conclusion:**

This case highlights the diagnostic complexity of abdominal pain in early pregnancy, particularly in patients with Crohn’s disease and anatomic variations such as intestinal malrotation.

## Introduction

Acute abdominal pain during pregnancy is a diagnostic and therapeutic challenge. Physiological changes in pregnancy, overlapping symptomatology, and concerns about fetal safety limit the use of certain diagnostic tools. When compounded with chronic gastrointestinal diseases like Crohn’s, the evaluation becomes even more complex. We report a rare case of a pregnant woman with Crohn’s disease who presented with an acute abdomen, ultimately diagnosed as perforated left-sided appendicitis in the setting of large bowel malrotation.

## Case report

A 36-year-old Gravida 4, Para 3, woman at 5 weeks and 6 days gestation (confirmed by ultrasound) presented to the emergency department (ED) with acute, progressive abdominal pain localized initially to the left lower quadrant, associated with nausea and three episodes of non-bloody, non-bilious vomiting. She had no fever, diarrhea, or urinary symptoms. Her past medical history was significant for Crohn’s disease (fistulizing perianal subtype) diagnosed in 2008, previously treated with infliximab, adalimumab, vedolizumab, and currently on Ustekinumab every four weeks. 

Her surgical history included three cesarean sections, multiple incision and drainage procedures for perianal abscesses, and a perianal fistula repair. Her most recent dose of Ustekinumab was administered 10 days prior to presentation. 

**Fig. 1:**
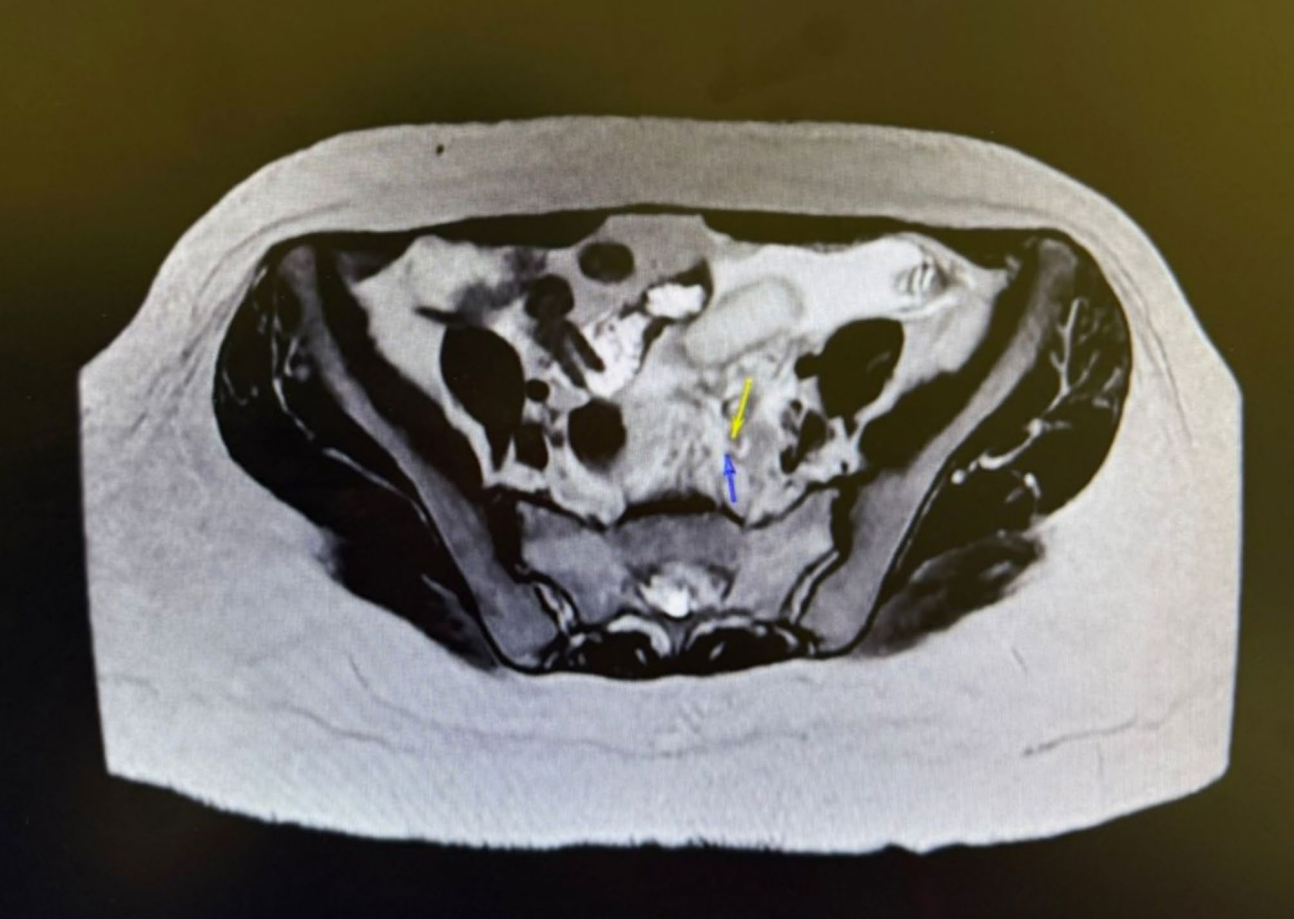
Axial cut MRI- demonstrating free fluid with a left-sided possible adaxial mass

On examination, she appeared well, with hemodynamic parameters within normal limits. The abdominal exam revealed generalized tenderness without peritoneal signs. The urine pregnancy test was positive, and the serum β-hCG measured 19,957 IU/L. Bedside ultrasound confirmed an intrauterine gestation with fetal cardiac activity as well as uncomplicated biliary stones. Laboratory studies demonstrated leukocytosis and elevated inflammatory markers (ESR 114 mm/hr, CRP 60 mg/L). An MRI abdomen was performed to assess possible causes of progressive abdominal pain, including a flare-up or complication of her IBD, the scan was inconclusive for any specific surgical pathology, but demonstrated free pelvic fluid and a suspected ruptured ovarian cyst vs. possible heterotopic pregnancy (Fig. 1). A Pelvic ultrasound confirmed a viable intrauterine pregnancy with fetal cardiac activity and normal appearing ovaries.

Despite pain management with intravenous opioids, her abdominal pain worsened, raising concern for an underlying surgical cause. Gynecology was consulted to assess the possibility of a heterotopic pregnancy and advised admission with repeat imaging and observation. General surgery was consulted, and a decision was made to proceed with a diagnostic laparoscopy due to continued pain and diagnostic uncertainty.

Intraoperatively, the surgical team identified a malrotated large bowel, with the cecum and appendix located in the left iliac fossa. Multiple pockets of purulent fluid were noted throughout the peritoneal cavity, and a perforation was seen 1 cm distal to the base of the appendix. Adhesiolysis was performed, and the perforated appendix was resected using an endoscopic stapling device. Extensive abdominal lavage was carried out across all quadrants. A pelvic drain was placed.

Postoperatively, the patient did well. Pain improved steadily, she tolerated oral intake, ambulated well, and remained afebrile. No signs of intra-abdominal sepsis or pregnancy complications were noted. Laboratory markers normalized. She was discharged home in stable condition. Final surgical pathology confirmed acute perforated appendicitis.

## Discussion

The evaluation of acute abdomen in pregnancy necessitates early recognition of potentially serious diseases [1]. Delayed or missed diagnosis of surgical emergencies such as appendicitis can result in maternal sepsis, uterine irritability, preterm labor, and fetal loss. Appendiceal perforation, which occurs more frequently in pregnant patients due to diagnostic delays, significantly increases fetal mortality—reported as high as 36% in some series [2].

Laparotomy in advanced gestation carries risks of uterine injury, preterm labor, and anesthetic complications. Conversely, diagnostic laparoscopy in early pregnancy has been shown to be relatively safe although previously thought to be associated with higher fetal complications [3]. More recent reviews have found similar fetal outcomes for both open and laparoscopic techniques, with likely superior surgical outcomes using laparoscopy [4].

Appropriate imaging in the diagnosis of appendicitis has resulted in decreased negative appendectomy rate from as high as 25% to approximately 1–3% [5].

The table below summarizes recommended imaging/diagnostic modalities when considering appendicitis in pregnancy, their diagnostic performance, and pregnancy-related considerations (Table 1).

**Table 1:** The performance characteristics of different diagnostic modalities in early pregnancy to diagnose appendicitis

Modality	Ability to visualize appendix (%)	Radiation	Sensitivity (%)	Specificity (%)	Indication/use case	Drawbacks in pregnancy
Ultrasound (US)	3–64	none	80–90 (variable)	80–95	First-line for fetal viability, ectopic pregnancy, gynecologic causes, gallbladder, and hydronephrosis [5, 6]	Operator dependent, decreased visualization chance with each trimester, not tolerated well with peritonitis.
Magnetic Resonance Imaging (MRI)	60–80	none	96.8–100	93–99	Alternative when US is inconclusive; preferred for appendicitis, bowel pathology, soft tissue masses [6, 7]	Expensive; motion artifacts; less available
Computed Tomography (CT)	85–94	+	89–100	90–99	Reserved for equivocal cases; superior for retroperitoneal, ureteric, or complex intra-abdominal pathology [7–9]	Lifetime cancer risk; increase miscarriage rate on multiple CT for the first trimester.
Diagnostic laparoscopy	100	none	Definitive	Definitive	Used when imaging is inconclusive or deterioration occurs; provides direct visualization and therapeutic intervention [7, 10]	Invasive; requires anesthesia; potential fetal risk.

Diagnosing surgical causes of acute abdomen in pregnancy is fraught with challenges due to physiological and anatomic changes. Appendicitis, the most common non-obstetric surgical emergency in pregnancy, may present atypically [1]. As the uterus enlarges, the appendix can be displaced cephalad, leading to pain in unusual locations. Moreover, leukocytosis and mild abdominal discomfort can occur in normal pregnancy, obscuring clinical signs. Also pregnancy has been shown to cause appendicitis due ectopic uterine decidual tissue in rare cases [11, 12].

In this case, the diagnostic process was further complicated by coexisting Crohn’s disease and incidental discovery of an intestinal malrotation. Malrotation, attributed to abnormal midgut development during embryogenesis, occurs rarely in adults, but can alter expected anatomic landmarks, further misleading imaging interpretations [13]. It has been reported with an incidence of approximately 0.04% in adults and may result in left-sided appendicitis due to ectopic cecal positioning. Most symptomatic adults with intestinal malrotation present with bowel ischemia or bowel obstruction secondary to midgut or cecal volvulus; however, a minority of patients may present with acute left-sided appendicitis. Left-sided appendicitis has also been described in cases of situs inversus or with an unusually long appendix [13, 14].

Patients with Crohn’s disease presenting with acute abdominal pain pose a specific diagnostic challenge due to multiple possible etiologies related to active disease (gastrointestinal stenoses/strictures, abscesses, and fistulae) or complications from chronic inflammation (bacterial infection, malignancy), or complications from previous IBD-related surgeries (obstruction, bacterial overgrowth, hernias) and extraintestinal manifestations of IBD that result in abdominal pain (cholelithiasis, nephrolithiasis, and pancreatitis) [15].

Noteworthy as well was the patient’s treatment with Ustekinumab. This monoclonal antibody to interleukin (IL) 12/23p40, used to treat moderate to severe IBD, suppresses immune responses and reduces inflammation. It has been reported to cause appendicitis in real world drug monitoring [16].

The diagnostic approach to imaging in pregnancy requires careful consideration based on presumed etiology of the cause of pain, while weighing the risks of exposure to ionizing radiation. MRI is the preferred modality for abdominal imaging in pregnancy due to its safety and high visualization rate of the appendix. Its demonstrates superiority to CT in its ability to visualize soft tissue structures with no contrast, with gadolinium appearing to add little to its diagnostic utility for abdominal pain [5, 6]. Ultrasound with graded compression is the preferred initial imaging modality for suspected appendicitis in pregnant women, its sensitivity and specificity performance appears similar between pregnant and non-pregnant patients, but has low visualization rates, which worsens with every trimester [5, 17].

CT with contrast has the highest diagnostic accuracy but is typically reserved for when urgent information is required, such as traumatic injury and other imaging modalities are insufficient in providing a diagnosis, but requires careful risk consideration due to fetal radiation exposure, particularly in the first trimester [10, 11]. In cases with diagnostic uncertainty and concerns for an acute abdominal process, diagnostic laparoscopy or laparotomy both been demonstrated safe and effective, with recent meta-analysis demonstrating non-inferiority of laparoscopy with respect to pregnancy outcomes but superiority with regards to surgical outcomes when compared [4, 10].

This challenging diagnostic case, uniquely described in the literature, highlights the diagnostic considerations in early pregnancy, with additional complexity due to chronic gastrointestinal disease and incidental congenital variation. Emphasizing the importance of multidisciplinary coordination to enable timely surgical intervention and prevent further maternal-fetal morbidity.

## Conclusion

This case underscores the complexity of evaluating acute abdominal pain in early pregnancy, particularly in patients with Crohn’s disease and congenital anomalies like intestinal malrotation. Early surgical consultation and a low threshold for diagnostic laparoscopy are critical when initial investigations are inconclusive.

## Data Availability

No datasets were generated or analysed during the current study.
